# Outcomes of an HIV cohort after a decade of comprehensive care at Newlands Clinic in Harare, Zimbabwe: TENART cohort

**DOI:** 10.1371/journal.pone.0186726

**Published:** 2017-10-24

**Authors:** Tinei Shamu, Cleophas Chimbetete, Sandra Shawarira–Bote, Tinashe Mudzviti, Ruedi Luthy

**Affiliations:** 1 Newlands Clinic, Highlands, Harare, Zimbabwe; 2 School of Pharmacy, University of Zimbabwe, Mount Pleasant, Harare, Zimbabwe; University of Washington Department of Global Health, UNITED STATES

## Abstract

**Background:**

Data on long-term outcomes of patients receiving antiretroviral therapy (ART) in sub-Saharan Africa are few. We describe outcomes of patients commenced on ART at Newlands Clinic between 2004 and 2006 after ≥10 years of comprehensive care including, psychosocial, adherence and food support.

**Methods:**

In this retrospective cohort study, patient data from an electronic medical record collected during routine care were analysed. We describe baseline characteristics, virological and clinical outcomes, attrition rates, and treatment adverse effects until November 2016. We defined virological suppression as viral load <50 copies/ml and virological failure as >1000 copies/ml after ≥6 months of ART.

**Results:**

We analysed data for 605 patients (67% female) who commenced ART, and were followed-up for 5819 person-years (median: 10.7 years, IQR: 10.1–11.4). Median age at ART initiation was 34 years (IQR: 17–42). Pre-ART, 129 (21.3%) patients had history of pulmonary tuberculosis (PTB). In care, 66 (11%) developed PTB, and 24 (4%) developed extrapulmonary tuberculosis. 385 (63.6%) patients experienced ≥1 adverse event, the most frequent being stavudine-induced peripheral neuropathy (n = 252, 41.7%). At database closure on 14 November 2016, 474 (78.3%) patients were still in care, 428 (90.3%) being virologically suppressed, and 21 (4.4%) failing. While 483 (79.8%) remained on first line, 122 (20.2%) were switched to second line ART. Fifty-nine patients (9.8%) were transferred to other ART facilities, 45 (7.4%) were lost to follow-up, 25 (4.1%) died, and two stopped ART.

**Conclusion:**

Comprehensive HIV care can result in low mortality, high retention in care and virologic suppression rates in resource limited settings.

## Introduction

Zimbabwe, with a population of 12.9 million is among countries in the sub-Saharan region experiencing a mature HIV epidemic with a decline in prevalence over the last decade from 27.2% in 1998 to 14.6% in the 15–64 year age group in 2016, whilst HIV incidence stabilized at 0.45% in the same age group [1]. The majority of people living with HIV (PLHIV) in 2016 were women, with a prevalence of 16.7% and annual incidence of 0.59% while men had a lower prevalence (12.4%) and annual incidence (0.31%). The epidemic is mostly heterosexual and the risk factors include migration, high-risk sex and key populations. HIV treatment sites increased from 530 in 2010 to 1459 sites in 2014 [2]. Coverage of antiretroviral therapy (ART) has increased from 5% in 2004 to 83% in 2011 with widespread ART roll-out, and 86.8% in 2016 [1,2].

Over the past decade, access to ART has significantly improved in resource limited countries such as Zimbabwe [2]. Short and medium term studies from sub-Saharan Africa show that despite high early mortality rates due to late presentation, substantial loss to follow-up, and high rates of drug substitution due to toxicity, antiretroviral treatment programs have achieved outcomes comparable to those in developed settings especially in terms of CD4 count gains and viral suppression [3–6]. However, these studies were done for a short duration and were unable to account for outcomes of patients as they stayed longer on treatment [7–11]. Assessment of an ART programme is essential to establish its successes, failures and lessons learnt.

From its inception in 2004, Newlands Clinic became part of the coordinated public-private partnership in the Ministry of Health. The clinic is contributing to the national ART response by providing a comprehensive care and treatment programme for poor PLHIV. As of October 2016, approximately 6,000 patients (63% of them women) with HIV infection were receiving care at the clinic [12]. A description of long-term outcomes from a well-characterized cohort in Zimbabwe has not yet been undertaken. The objective of this analysis was to evaluate the long-term outcomes in this cohort of patients who were recruited into care and commenced on ART by 31 December 2006. Specifically, we assessed four outcome indicators: attrition, virological suppression, immune-recovery and clinical outcomes of this cohort.

## Methods

### Definition of terms

**Baseline**–time of ART commencement;

**Last CD4 count or viral load**–the latest CD4 count or viral load before data abstraction on the 14^th^ of November 2016 for patients who were still in care at that time.

### Study design

We conducted a retrospective cohort study in which routinely collected data of patients recruited into care and initiated on ART at Newlands Clinic between 1 March 2004 and 31 December 2006 were analysed. The censoring date for data abstraction was 14 November 2016.

### Study setting

Newlands Clinic was founded in Harare, Zimbabwe in February 2004 with the aim of providing comprehensive ART services to PLHIV from poor communities in and around Harare. It was established on a nurse-led model of care in which nurses with counselling qualifications provide routine adherence and psychosocial counselling at each clinic visit, and doctors support the nurses with clinical decisions including initial patient evaluation, where a diagnosis needs to be made and provision of prescriptions. Patients requiring more specialised mental health support are referred to a psychologist who is also part of the staff at the clinic. This comprehensive care, apart from provision of ART medicines also includes food supplementation for patients failing to secure food for their households as well as the underweight as determined by body mass index (BMI) <18.

Newlands Clinic is a private voluntary organisation (PVO) that is largely private funded by donations sourced through the Swiss based Ruedi Luethy Foundation and the Swiss Agency for Development and Cooperation (SDC). The Zimbabwean government supports the clinic through provision of antiretroviral and anti-TB medicines as well as HIV and syphilis test kits that it distributes via the national pharmaceutical logistics unit (NatPharm).

Newlands Clinic prioritised the recruitment of patients from the poorest communities in and around Harare and Chitungwiza. Newlands Clinic funded all medical costs for patient management provided at the clinic including consultation fees, laboratory tests and limited outsourced services including radiology and imaging. In collaboration with other non-government organisations (NGOs) like Christian Care and World Food Programme (WFP), patients from vulnerable homes received food support.

During the period of March 2004–December 2006, the Newlands Clinic criteria for ART commencement included patients whose CD4 counts were <200 cells/mm^3^, pregnant women or patients who developed a WHO stage 3 or 4 opportunistic infection (OI). Antenatal services were not provided at the clinic, but pregnant women were referred to their local council polyclinics for those services.

Patients on first line ART received two nucleos(t)ide reverse transcriptase inhibitors (NRTIs) backbone and one non-nucleoside reverse transcriptase inhibitor (NNRTI) which was either nevirapine or efavirenz. During the study period, the preferred first line regimen for adult patients was stavudine/lamivudine/nevirapine, which was changed in 2007 to zidovudine/lamivudine/nevirapine, then in 2010 to tenofovir/lamivudine/nevirapine and finally in 2013 to tenofovir/lamivudine/efavirenz [13–15].

Patients on second line treatment received two NRTIs and a ritonavir boosted protease inhibitor (PI). During the study period, lopinavir/ritonavir was the preferred PI for second line treatment and was replaced in the 2013 national guidelines review which saw atazanavir/ritonavir becoming the preferred PI for second line treatment of adults [14]. Patients on each line of treatment were switched in accordance with the changes in national guidelines in most instances as supplies of medicines from NatPharm encouraged harmonization of regimens according to recommendations. However, the clinic discontinued stavudine use earlier than the national guidelines recommended so opting for zidovudine as guided by its observation of high stavudine related adverse events.

Treatment monitoring using HIV RNA viral loads at Newlands Clinic commenced in August 2013. Since then, patient viral loads were measured every six months for patients that were classified as being stable on ART. For those that did not achieve virological suppression, intensified adherence counselling was done for six weeks and the viral load was measured three months from the last unsuppressed viral load. According to the clinic’s routine practice, patients with viral load measurements above 500 copies/ml were considered to be failing, while those below 50 copies/ml were considered as having virological suppression. In this study, we defined virological suppression as viral load levels <50 copies/ml, low level viraemia as 50–1000 copies/ml and virological failure as >1000 copies/ml. Virological and immunological outcomes were determined using the most recent viral load and CD4 count respectively for each patient who was still in care by 14 November 2016.

Prior to HIV RNA viral load monitoring, the clinic used a boosted p24 antigen test as the monitoring test for virological suppression in conjunction with CD4 and clinical monitoring for immunological and clinical failure respectively. P24 antigen levels below 5pg/ml were considered as virological suppression while two measurements >5pg/ml with intensified adherence counselling in between was considered as virological failure.

Patients within the clinic’s care were assigned an activity status in which active patients were those currently in care; lost to follow-up were those who did not attend their last scheduled visit and could not be contacted for over 120 days thereafter. Deceased patients were those whose death had been communicated to the clinic either by evidence of a death certificate or information from their next of kin; transferred out were those that were provided with an official transfer letter to a different ART services facility; and those otherwise inactive would have stopped ART and withdrawn from care.

Diagnosis of adverse effects was done by qualified doctors. Zidovudine induced anaemia was defined as reduction in haemoglobin to <8g/dL after initiation of a zidovudine containing ART regimen in individuals who had normal haemoglobin levels at the time of zidovudine commencement (Female:11.5 – 16g/dL; Male: 13.2 – 18g/dL). Peripheral sensory neuropathy diagnosis was made clinically when patients on ART presented with burning sensation in the feet with or without altered sensation. Lipodystrophy diagnosis was also made clinically when patients presented with signs of abnormal fat redistribution with fat deposits on the neck, abdomen, breast and back of upper trunk accompanied by loss of fat in the face, buttocks, arms and legs.

### Data collection

Data were collected into an electronic medical record during patient management including demographic and clinical findings. Customised queries were written in Microsoft SQL Server Management Studio 2012 to abstract this data using the inclusion and exclusion criteria at query level to obtain only the data from patients of interest. The query outputs were saved in Microsoft Excel. Stata version 12.1 (StataCorp LP, Texas, USA) was used to import the data from Microsoft Excel for analysis S1 File shows the dataset analysed in this study.

We included all eligible participants in the database who were enrolled into the cohort and commenced on ART between 1 March 2004 and 31 December 2006 with more than one clinic visit after ART commencement. We excluded participants who were ART experienced upon recruitment into Newlands Clinic care.

### Statistical analysis

We used descriptive statistics including frequencies, proportions, medians and interquartile ranges to describe baseline characteristics, immunological, virological and clinical outcomes.

### Ethical considerations

The study was approved by the Newlands Clinic Research Unit and the Medical Research Council of Zimbabwe (MRCZ Approval No. MRCZ/E/149). Newlands Clinic is part of the International epidemiologic Databases to Evaluate AIDS–Southern African Region (IeDEA-SA). Patients whose data were used in this study completed an informed consent form, which allowed for the data collected during their routine care to be used in research studies.

## Results

### Baseline characteristics

A total of 605 patient records were analysed, 404 (67%) being female. Patients were followed up for 5819 person-years from time of ART initiation. The median follow-up time was 10.7 years (IQR: 10.1–11.4) since ART initiation. Patients were initiated on ART after a median of five weeks from enrolment (IQR: 3–13). Table 1 summarises the baseline demographic and clinical characteristics stratified by sex. The median baseline age was 34 (IQR: 17–42) years, with 71.7% of patients aged ≥25 years.

The majority of patients (n = 388, 64%) presented at the initial visit with WHO stage 3 or 4 defining illnesses.

**Table 1:** Baseline demographic and clinical characteristics (N = 605).

	Frequency n (%)^a^		
	Male	Female	All
**Demographics**			
Study participants	201 (33.2)	404 (66.8)	605 (100)
Age at enrolment–Median (IQR)	34 (12–40)	35 (27–42)	34 (17–42)
Age Groups (years)			
0–4	11 (5.5)	17 (4.2)	28 (4.6)
5–12	44 (21.9)	42 (10.4)	86 (14.2)
13–24	22 (11.0)	33 (8.2)	55 (9.1)
25–40	74 (36.8)	193 (47.8)	267 (44.1)
41–65	49 (24.4)	118 (29.2)	167 (27.6)
>65	1 (0.5)	1 (0.2)	2 (0.3)
**Clinical Factors**			
WHO stage 1	10 (5.0)	38 (9.4)	48 (8.0)
WHO stage 2	55 (27.4)	113 (28.0)	168 (27.8)
WHO stage 3	107 (52.2)	191 (47.4)	298 (49.3)
WHO stage 4	29 (14.4)	61 (15.1)	90 (14.9)
**CD4 Count^b^ (cells/mm^3^) at enrolment**			
Median (IQR)	108(46–183)	127 (62–197)	121 (57–195)
≥500	12 (6.0)	13 (3.2)	25 (4.1)
350–499	7 (3.5)	24 (6.0)	31 (5.1)
200–349	33 (16.4)	73 (18.1)	106 (17.5)
<200	149 (74.1)	294 (72.8)	443 (73.2)
History of Pulmonary Tuberculosis	48 (23.9)	81 (20.1)	129 (21.3)
History of Extrapulmonary Tuberculosis	6 (3.0)	14 (3.5)	20 (3.3)
History of Cryptococcal Disease	1 (0.5)	6 (1.5)	7 (1.6)
History of Kaposi’s Sarcoma	0	2 (0.5)	2 (0.3)

^a^Unless where otherwise stated.

^b^CD4 counts for patients aged ≥ 5 years.

Over a fifth (n = 129, 21%) of participants had history of pulmonary tuberculosis prior to enrolling at Newlands Clinic while 20 (3%) had history of extrapulmonary tuberculosis. History of cryptococcal disease was uncommon with only seven patients (2%) having been diagnosed with cryptococcal meningitis prior to enrolling into Newlands Clinic. The median CD4 count for the seven patients with history of cryptococcal disease at baseline was 84 cells/mm^3^. Two female patients had a history of Kaposis’s Sarcoma at the time of their baseline visit.

### Treatment adverse effects

Treatment adverse effects were common with 385 (64%) patients having experienced at least one adverse event during follow-up. The most frequent adverse effect was stavudine induced peripheral sensory polyneurophathy (n = 252, 42%), followed by lipodystrophy, lipoatrophy and zidovudine induced anaemia. Table 2 summarises the frequencies of adverse events experienced by patients during follow-up.

**Table 2:** Frequency of treatment adverse effects experienced during follow-up (N = 605).

Adverse Effect	Frequency n (%)		
	Male	Female	Total
Stavudine related Peripheral Sensory Polyneuropathy	72 (35.8)	180 (44.6)	252 (41.7)
Lipodystrophy/Lipoatrophy	20 (10.0)	94 (23.3)	114 (18.8)
AZT induced Anaemia	4 (2.0)	18 (4.5)	22 (3.6)
Other Adverse event	20 (10.0)	23 (5.7)	43 (7.1)

AZT = Zidovudine

### ART duration and regimens

Patients were on ART for a median time of 10.7 years (IQR: 10.1–11.4). A fifth of the patients were transferred from first line to second line regimen during follow-up (n = 122, 20%), while the majority (n = 483, 80%) remained on first line regimen until time of analysis or exit from the cohort. Among 474 patients still in care at analysis, 363 (76.6%) were still receiving first line regimen while 111 (23.4%) were receiving second line regimen. None of the patients had been switched to a third line regimen by the end of follow-up. Among the same 474 patients, the median duration of first line was 10.7 years (IQR: 10.1–11.4), while the median duration of second line was 6 years (IQR: 2.3–8.7).

### Viral suppression

Of the 605 patients, 502 (83%) had HIV RNA viral loads measured at a median time of 10.6 years on ART (IQR: 10.2–11.3). Of the 474 patients that were still in care by end of follow-up, 428 (90%) had undetectable viral load levels on the last measurement before censoring.

### Immunological outcomes

After a median follow-up time of 10.2 years of ART (IQR: 9.8–11.1), the median of the last CD4 count measured was 477 (IQR: 336–637) cells/mm^3^ among patients that were still in care before censoring.

### Comorbidities

Of the 129 patients who had history of pulmonary tuberculosis at baseline, 19 (15%) experienced a second episode. Overall, 66 (11%) patients developed pulmonary tuberculosis while in care. Twenty-four (4%) patients were diagnosed with extrapulmonary tuberculosis while in care.

Among the patients who did not present with a history of cryptococcal disease, ten developed cryptococcal meningitis during follow-up. Of these, two were diagnosed with cryptococcal meningitis before starting ART, while four were diagnosed within the first twelve weeks of ART. One patient experienced a second episode 57 weeks after the first diagnosis. Among the seven who had history of cryptococcal disease at initial visit, two experienced second cryptococcal disease episodes, one at week 409 of ART and the other at weeks 70 and 206 of ART.

Fourteen patients were diagnosed with malignancies during follow-up. The most commonly diagnosed malignancy was cancer of the cervix (n = 6) followed by Kaposi’s sarcoma (n = 3). The remaining five patients each had a different form of malignancy. Overall, ten of the patients diagnosed with malignancies were still in care at the time of analysis, three were deceased and one lost to follow-up.

At the time of analysis, 13 (2%) patients (six male and seven female) had been diagnosed with diabetes mellitus and 120 (20%) with hypertension (26 (13%) male; 94 (23%) female). The median age at diagnosis of hypertension was 48 years (IQR: 41–54).

### Retention in care

Of the 605 patient files reviewed, 474 (78.4%) were still in care at the time of analysis (14 November 2016) while 25 (4.1%) had deceased and 45 (7.4%) lost to follow-up. The lowest retention in care rate was among patients aged 12–24 years at baseline with 36 out of 55 (65.5%) being in care at analysis. Table 3 summarises the attrition rates stratified by ART baseline age.

**Table 3:** Attrition rates as at time of analysis stratified by baseline age groups (N = 605).

Age group (years)	Enrolled	Patient Status n (%)^a^				
		Active	Deceased	LTFU	Stopped ART	Transferred Out
<5	28	26 (92.9)	1 (3.6)	0	0	1 (3.6)
5–11	86	71 (82.6)	5 (5.8)	2 (2.3)	0	8 (9.3)
12–24	55	36 (65.5)	1 (1.8)	7 (12.7)	0	11 (20.0)
25–40	267	205 (76.8)	10 (3.8)	25 (9.4)	1 (0.4)	26 (9.7)
41–65	167	135 (80.8)	8 (4.8)	11 (6.6)	1 (0.6)	12 (7.2)
>65	2	1 (50.0)	0	0	0	1 (50.0)
Total	605	474 (78.4)	25 (4.1)	45 (7.4)	2 (0.3)	59 (9.8)

^a^Row percentages

LTFU = Lost to follow-up

Among the 25 deceased patients, nine were male and 16 were female. Table 4 shows the time of death, loss to follow-up, transfer out and stopping ART as measured since ART commencement. The median duration on ART among deceased patients was 8.7 years (IQR: 7.2–9.8). The cause of death for nine of the patients was unknown and the death certificates were not provided. Among those with known causes of death, the most frequent were extrapulmonary tuberculosis (n = 3) and liver failure (n = 2). The remaining 11 each had different causes of death namely liver cirrhosis, pulmonary tuberculosis, abdominal malignancy, cervical cancer, pancreatic cancer, hypertensive heart disease, chronic renal failure, cryptococcal meningitis, diabetic ketoacidosis, dysentery, and bacterial pneumonia. The first year of ART had the highest proportion of patients lost to follow-up, while the highest proportion of transfers to other facility were in the first two years of ART.

**Table 4:** Time to death, loss to follow-up, transfer out or opting out of care in years since ART commencement (n = 131).

	Frequency n (%)				
ART Year	Deceased	LTFU	Transferred	Opted out	Total
**0**	1 (4.0)	17 (37.8)	10 (16.9)	1 (50.0)	29 (22.1)
**1**	3 (12.0)	4 (8.9)	10 (16.9)	-	17 (13.0)
**2**	-	7 (15.6)	9 (15.2)	-	16 (12.2)
**3**	2 (8.0)	6 (13.3)	7 (11.9)	-	15 (11.5)
**4**	-	3 (6.7)	4 (6.8)	-	7 (5.3)
**5**	-	1 (2.2)	1 (1.7)	-	2 (1.5)
**6**	-	1 (2.2)	2 (3.4)	-	3 (2.3)
**7**	3 (12.0)	-	4 (6.8)	-	7 (5.3)
**8**	4 (16.0)	-	4 (6.8)	-	8 (6.1)
**9**	6 (24.0)	5 (11.1)	4 (6.8)	-	15 (11.5)
**10**	4 (16.0)	1 (2.2)	3 (5.1)	1 (50.0)	9 (6.9)
**11**	2 (8.0)	-	1 (1.7)		3 (2.3)
**Total**	25 (100)	45 (100)	59 (100)	2 (100)	131 (100)

## Discussion

To our knowledge this is one of the first comprehensive analysis of outcomes of a in a well-characterized African ART cohort outside a research setting with ≥10 years of follow up. Overall, we observed that our cohort consisted of young, predominantly female and severely immunosuppressed individuals, representative of African ART patients at the start of the ART rollout. The population in this cohort started ART at very advanced stage of disease, with 64% of the patients being in WHO stages 3 or 4, and with a median CD4 count of 121 cells / mm^3^ (IQR: 57–195). We observed that the overall retention and viral suppression rates were high with remarkable immunologic recovery after ≥10 years of follow-up. The mortality rate was very low. The majority of patients (80%) remained on first line ART. These findings are encouraging given the context of an initially highly immune-deficient population. Tuberculosis was the commonest opportunistic infection diagnosed in this cohort and the commonest ART side effect reported was stavudine related sensory peripheral neuropathy.

The overall rate of loss to follow-up was very low with most patients having been lost to follow-up during their first year of ART (n = 17, 37.8%), but this may not reflect the attrition rates observed in other settings [4]. This rate is much lower than retention rates reported in the Zimbabwe national OI / ART program [1]. Newlands Clinic has developed a robust mechanism of following up patients who miss their scheduled visits. The treating nurse initiates follow-up on the same day a patient misses their visit through a phone call. Furthermore, each nurse looks after a specific group (cohort) of patients. This helps establish a very good nurse-patient relationship.

The clinic offers a comprehensive care package to patients, which includes food assistance and assistance with school fees for poor children. Treatment and care is offered at no cost to the patient. We believe that all these services help to retain patients in care. The reported level of retention may not be achieved in routine busy settings where resources are minimal and health care workers are overwhelmed, but we do demonstrate that with a dedicated clinical team and routine viral load monitoring high retention in care can be attained. Our mortality rate of 4% is much lower than what has been reported in similar cohorts [16]. A similar cohort in Haiti reported a 10 year mortality rate of 27% among patients who commenced ART in 2003 and 2004 [17]. The very low mortality rate can be attributed to the availability of excellent monitoring for ART treatment success and toxicity. Newlands Clinic has more resources to offer care that compared to public health facilities. Resources enable more accurate diagnosis and treatment of life threatening illnesses.

Among patients still in care after 10 years of ART, HIV RNA suppression rate of 90% was very good. We believe this is an impressive accomplishment compared to the set target, as well as to other programmes in sub-Saharan Africa [18,19]. Our results are also encouraging in view of the 90-90-90 target set by UNAIDS. In addition, after ≥10 years of care, only 20% of patients had been switched to second-line and no patient had been switched to third line treatment, highlighting the durability of existing regimens available in resource-limited settings. This observation of high viral suppression is additionally important from the perspective of HIV prevention and underscores the gains of treatment as prevention. Furthermore, there was a significant immunological recovery with CD4 cell count rising from a baseline median of 121 cells/mm^3^ to 477 cells/mm^3^ among patients that were still in care at data abstraction after a median follow-up of 10.7 years. However, this median CD4 count at the end of follow-up remains lower than the normal ranges. Previous studies have shown that despite effective ART, complete CD4 cell recovery occurred in very few individuals and was associated with baseline CD4 T-cell count and viral load suppression [20].

More than half of the patients required at least one drug substitution while on first line; this was mostly due to the inclusion of stavudine in the majority of the ART regimens prescribed at treatment start. Most of the substitutions due to toxicity were attributable to stavudine toxicity i.e. lipodystrophy/lipoatrophy and peripheral sensory neuropathy. These findings are similar to what has been reported in a Ugandan cohort [21].

This evaluation does have some limitations. A more rigorous tracing of patients lost to follow-up, particularly during the first year may have shed more light into the true status of each of those 17 patients lost to follow-up. The clinic did not offer routine viral load testing before 2013 and hence we could not report baseline viral load status in this analysis. This evaluation also has notable strengths, particularly the large enrolment of the program and lengthy follow-up, which provide statistical strength to reinforce our findings.

## Conclusion

Comprehensive HIV care can result in low mortality, high rates of retention in care and virologic suppression in resource-limited settings.

## Supporting information

S1 File TENART DATASET SF.xlsx. TENART cohort dataset.
(XLSX)
